# Three-dimensional visualization of intrauterine conceptus through the uterine wall by tissue clearing method

**DOI:** 10.1038/s41598-017-06549-6

**Published:** 2017-07-20

**Authors:** Kyosuke Kagami, Yohei Shinmyo, Masanori Ono, Hiroshi Kawasaki, Hiroshi Fujiwara

**Affiliations:** 1 Department of Obstetrics and Gynecology, Graduate School of Medical Sciences, Kanazawa University, Ishikawa, 920-8640 Japan; 2 Department of Medical Neuroscience, Graduate School of Medical Sciences, Kanazawa University, Ishikawa, 920-8640 Japan; 3 Brain/Liver Interface Medicine Research Center, Kanazawa University, Ishikawa, 920-8640 Japan

## Abstract

Visualization of specific cells in the three-dimensional organ architecture is one of the key steps to develop our knowledge about pathophysiological mechanisms in various organs. In this study, we successfully obtained stereoscopic whole images of the intrauterine murine embryo and placenta through the uterus using a modified tissue clearing CUBIC method. By this procedure, we can recognize the three-dimensional relationships among various tissues within the pregnant uterus and analyze free-angle images of cross-sections with single-cell resolution using a computer system. Based on these data, we can select optimal cross-section angles and then produce the corresponding tissue slices that are adequate for further immunohistochemical examination. Furthermore, using transgenic mice, distinct images of an EGFP-positive embryo and the placenta can be obtained, confirming the precise three-dimensional location of invading trophoblasts in the feto-maternal interface in the uterus. These results indicate that this procedure will significantly contribute to analyzing pathophysiological mechanisms in reproductive organs.

## Introduction

The uterus is a crucial reproductive organ for pregnancy. Once fertilization has been established, a sequence of biological events leading to pregnancy such as implantation and the formation of the placenta proceed in the uterus^1^. During placentation, programed tissue remodeling takes place in the maternal endometrium and the volume of the uterus is markedly increased in accordance with fetal development and growth. In humans, extravillous trophoblasts invade maternal spiral arteries and replace both endothelial and muscle layers, inhibiting arterial contraction and enhancing maternal blood flow into the syncytiotrophoblast-lining intervillous spaces^2^. Accordingly, the interaction between embryo-derived trophoblasts and maternal immune cells is an inevitable process. It is well-known that the disturbance of this vascular remodeling process in early pregnancy will lead to the onset of preeclampsia in the later phase^3^. In addition, since the embryo is a semi-allograft for the mother, the immunotolerance of trophoblasts against maternal immune attack must be acquired during cell-to-cell semi-allograft interactions in order to achieve successful pregnancy^4^. Consequently, the spatiotemporal analysis of invading trophoblasts and maternal cells in the feto-maternal interface is a key step to understand the pathophysiological mechanisms of placentation^5^.

The feto-maternal interface exists not only at placental sites, but also along the fetal membrane that contains amniotic fluid with a spherical structure within the uterine cavity. Therefore, the stereoscopic visualization of these areas is one of the attractive approaches to investigate the spatiotemporal events during placentation. Previously, many studies reported three-dimensional (3D) structures of the pregnant uterus using classical tissue sections and histological techniques^6–10^. In these methods, a large number of sequential thin tissue sections were prepared and subjected to various kinds of staining methods. Although these classical techniques are useful for analyzing fine structures within tissue sections, the successful reconstruction of 3D whole images of the pregnant uterus is difficult because tissue sections are often deformed during cutting and staining.

Recently, several groups developed tissue clearing methods: Sca/e, SeeDB, CLARITY, 3DISCO, and CUBIC^11–15^. Using these methods, light-sheet laser scanning microscopy can reconstruct 3D transparent images of the organ structures, and also provide 2D images of free-angle sections without making tissue slices^16^. By combining these techniques and fluorescent staining, 3D structures of regional axon networks in the brain can be visualized, providing valuable information to analyze the pathophysiology of neuroscience.

Based on this background, we applied the currently-reported tissue clearing methods to the murine pregnant uterus. In this study, considering the structural characteristics of the uterus and placenta, we selected a CUBIC system as a tissue clearing protocol and successfully obtained stereoscopic 3D images of the intrauterine conceptus with single cell resolution. Furthermore, using EGFP transgenic mice, the improvement of contrast images of the embryo-derived cells was also investigated.

## Results

### Tissue clearing of pregnant uterus using CUBIC

**Figure 1:**
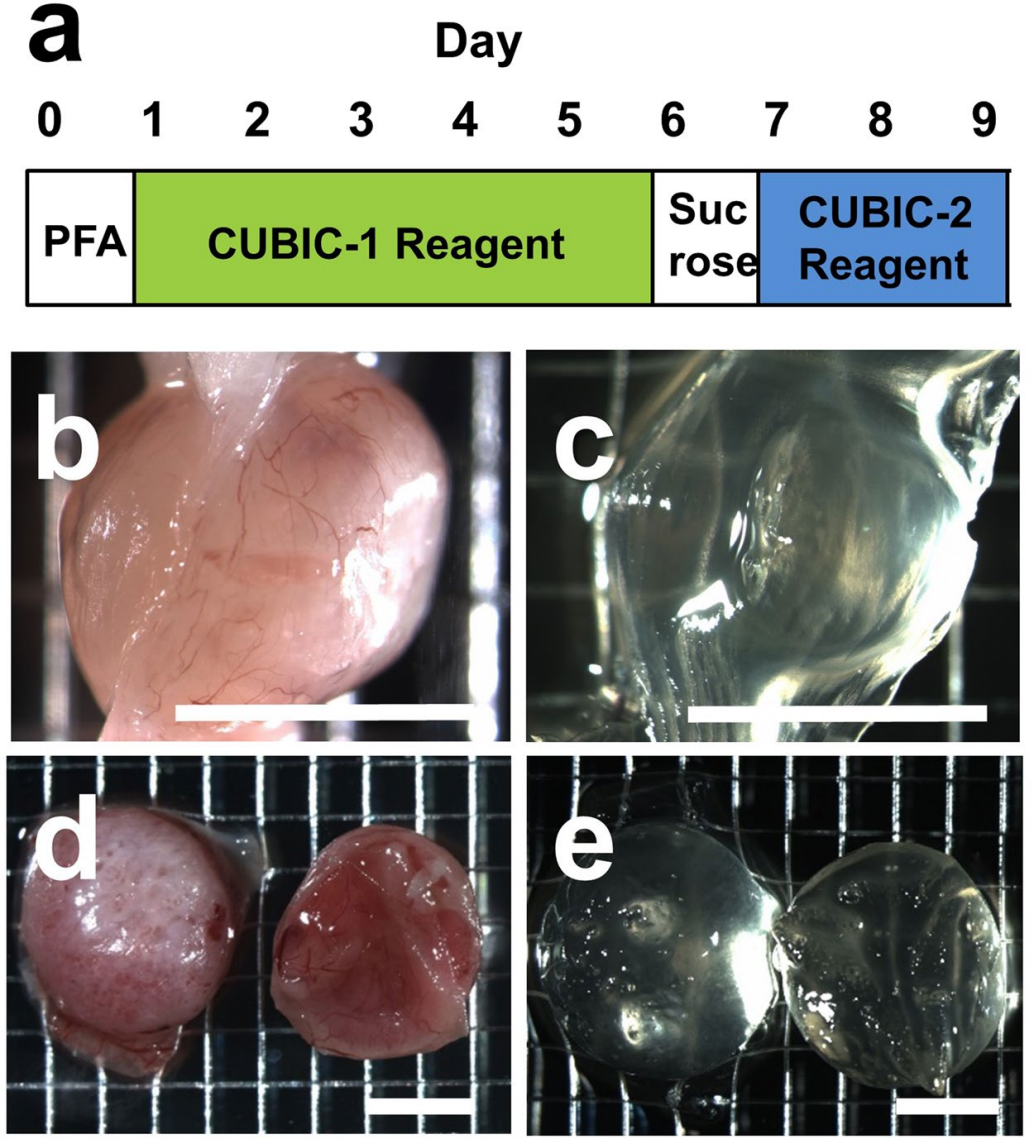
Tissue clearing of pregnant uterus and placenta. (**a**) The procedure of tissue clearing of the pregnant uterus and placenta using the CUBIC protocol. (**b**,**c**) Bright field images of the pregnant uterus of ICR mice at E9.5 before (**b**) and after (**c**) tissue clearing. (**d**,**e**) Bright field images of the placenta at E14.5 before (**d**) and after (**e**) tissue clearing. Grids of the background were transparently visible through tissues after CUBIC (**c** and **e**). Scale bars, 4 mm.

Since the uterus and placenta contain large amounts of myoglobin and hemoglobin, respectively, we chose a CUBIC system that has the marked advantage of facilitating the efficient decolorization of endogenous chromophores, such as hemoglobin and myoglobin, within tissues^17^. Considering the volume of the uterus and developing stages of the embryo and placenta, we selected pregnant uteri from embryonic day 9.5 to 14.5 (E9.5–E14.5), which were expected to be adequate for analysis of the events at the feto-maternal interface using tissue clearing methods. According to our modified CUBIC protocol, the pregnant uterus successfully became transparent without shrinking or expanding its organ volume (Fig. 1b and c, E9.5). Grids of the background were transparently visible through tissues after CUBIC treatment (Fig. 1b and c). A longer incubation time (i.e., 10 days) with CUBIC-1 reagent was required to make the pregnant uteri (E14.5) transparent because their size and hemoglobin content were greater than those of pregnant uteri in the early stage. By this treatment, all placental tissues isolated from pregnant mice (E14.5) were successfully treated to become transparent using this modified CUBIC protocol (Fig. 1d and e).

**Figure 2:**
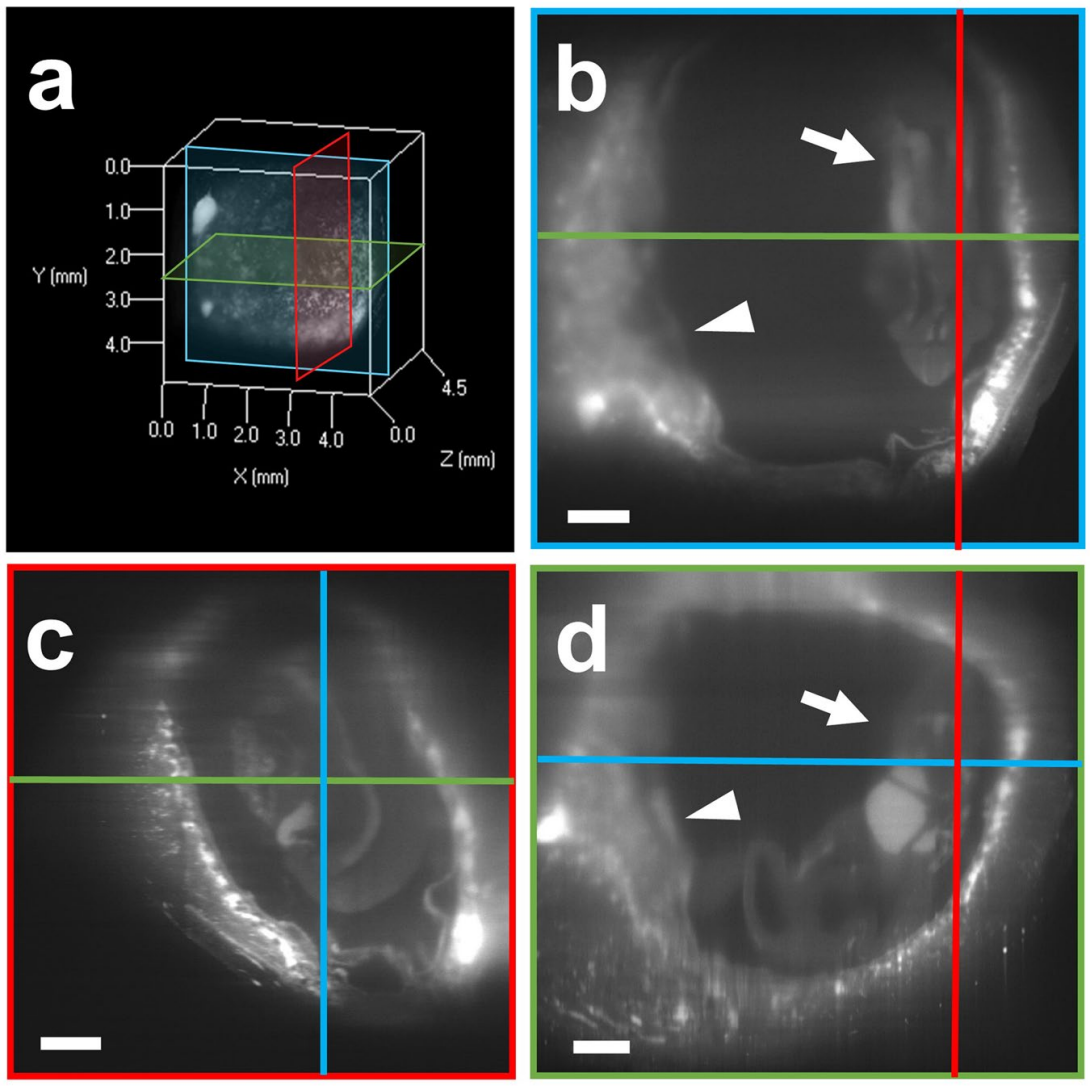
Intrauterine structures of the pregnant uterus visualized with a light-sheet microscope. The pregnant uterus at E11.5 was treated with CUBIC. After sequential X-Y cross-section images were obtained (**b**), 3D images were constructed (**a**), and other sectional images were reconstructed (**c** and **d**). A raw X-Y image (**b**, blue in **a**), reconstituted Y-Z image (**c**, red in **a**), and X-Z image (**d**, green in **a**) are shown. The placenta (arrowheads) and embryo (arrows) in the uterus are visible. An excitation wavelength of 638 nm was used for visualizing autofluorescence signals. Scale bars, 500 µm.

Then, to construct 3D images, we further analyzed the transparent uterine tissue block using a light-sheet microscope. After sequential X-Y cross-section images were obtained (Fig. 2b), we successfully obtained the whole image of the E11.5 embryo (Fig. 2, arrows) and placenta (Fig. 2, arrowheads) transparently through the uterine wall using autofluorescence (i.e., without any staining) (Fig. 2). These results suggest that our modified CUBIC method is appropriate to transparently observe structures of the murine pregnant uterus. In addition, based on these data, we can electronically reconstruct 3D images and/or cross-section images of the whole uterus from other angles (Fig. 2a,c,d).

### Transparent stereoscopic imaging of pregnant uterus using tissue clearing and nuclear staining

**Figure 3:**
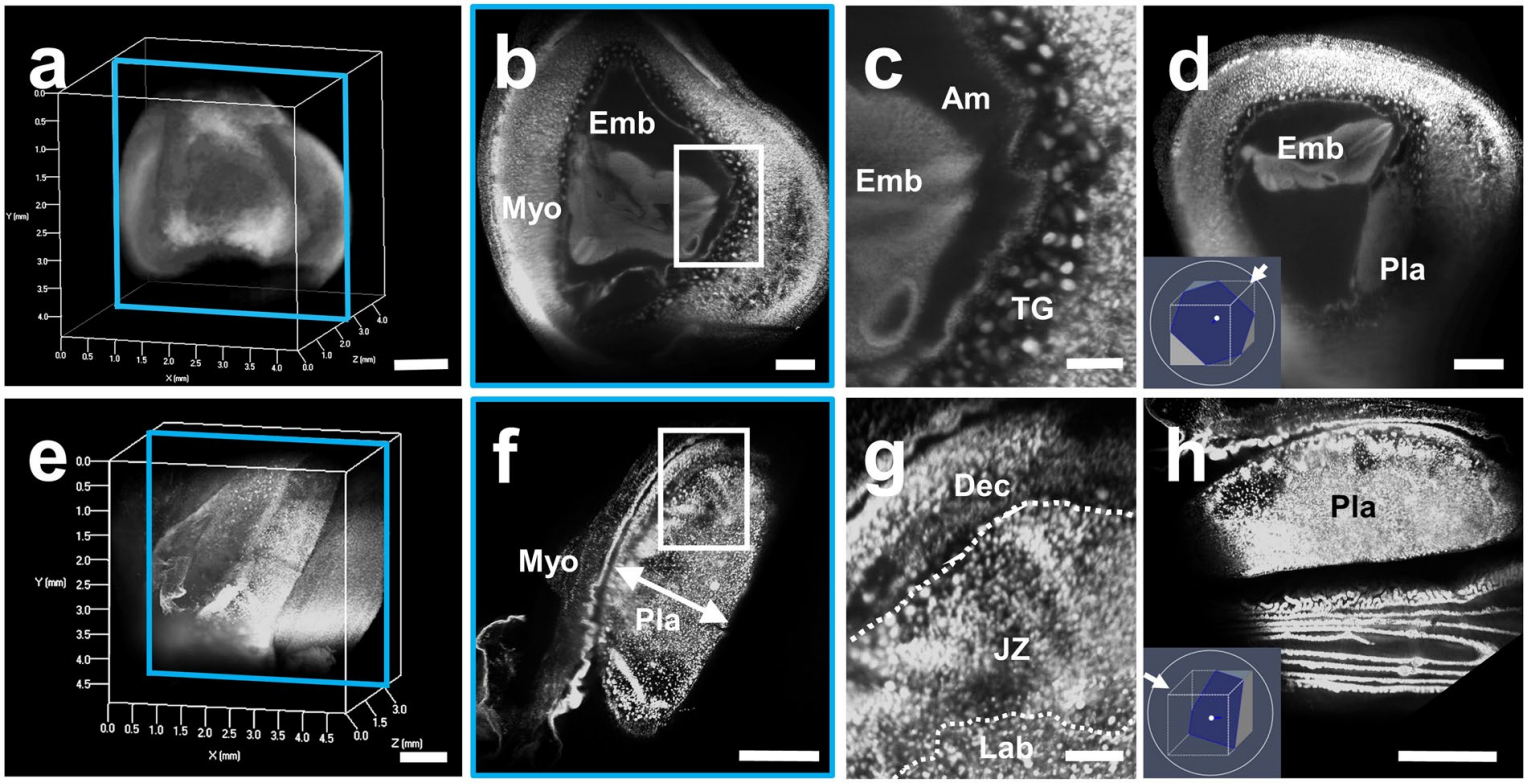
Three-dimensional and cross-sectional images of the pregnant uterus and placenta stained with PI. (**a**–**d**) Pregnant mice were transcardially perfused with 4% PFA and PI solution, and isolated pregnant uterus was subjected to CUBIC. Three-dimensional images were taken using a light-sheet microscope. Images of the pregnant uterus at E10.5 are shown. (**e**–**h**) After being isolated at E14.5, the placenta was immersed in PI solution, and 3D images were taken using a light-sheet microscope. Each organ is presented as a 3D image (**a** and **e**), a sectional image within a blue square of the left panel (b and f), a higher magnification image within a white square of the middle panel (c and g), and 2D images of angle-free cross-sections (**d** and **h**). Emb, embryo; Myo, myometrium; Dec, decidua; Am, amniotic cavity; TG, trophoblast giant cells; Pla, placenta; JZ, junctional zone; Lab, labyrinth zone. Scale bars, 1 mm (**a** and **e**), 500 µm (**b**,**d**,**f** and **h**), and 200 µm (**c** and **g**).

**Figure 4:**
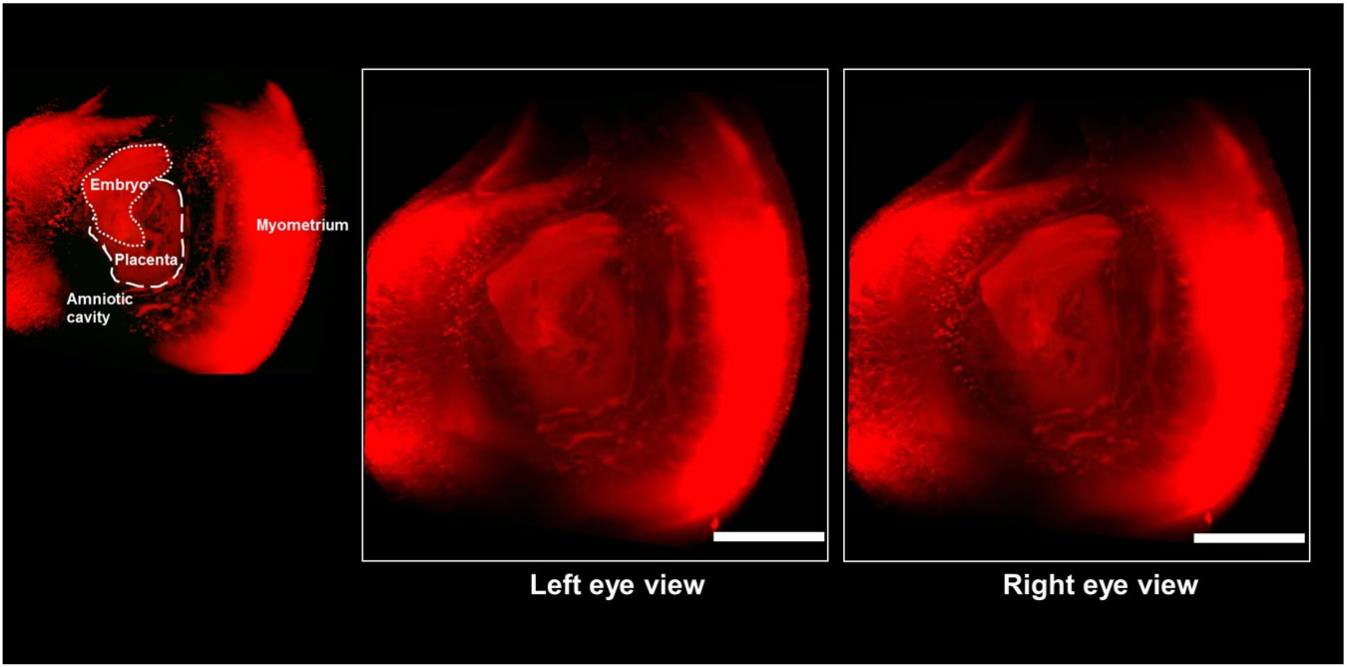
Stereoscopic image of the pregnant uterus stained with PI. Female mice were transcardially perfused with 4% PFA and PI solution, and isolated pregnant uterus was subjected to CUBIC. Three-dimensional images were taken using a light-sheet microscope, and stereoscopic images were reconstructed electronically. Scale bars, 1 mm.

Although macroscopic images of the embryo within the uterus were successfully obtained using autofluorescence, it remained difficult to detect detailed structures with single cell resolution. To visualize fine intrauterine structures, we performed fluorescent nuclear staining using propidium iodide (PI) (Fig. 3). When we immersed the tissue block in PI solutions according to the original staining method, PI signals at deep sites of the tissue block were faint. Consequently, we further added PI to the fixative solution with 4% PFA/PBS, which was transcardially perfused throughout the murine whole body. Due to this modification, we detected PI signals in the deep sites of the pregnant uterus and obtain sequential 2D images without making tissue sections, allowing the complete reconstruction of a transparent 3D image of both the pregnant uterus (Fig. 3a) and detached placenta (Fig. 3e). Based on these data, we can reconstruct 2D images from the angle-free cross-sections with single cell resolution (Fig. 3b,c,f and g: 2D images of X-Y cross-section, and Fig. 3d and h: 2D images of angle-free cross-section). These results indicate that fluorescent PI signals were not eliminated in the tissue clearing step by CUBIC Reagent-1, suggesting that our modified method is practical to analyze the pregnant uterus with this 3D visualization system. Consequently, we further acquired a transparent stereoscopic image reconstructed from binocular angles of 3D images, enabling us to visually understand the stereoscopic inner structure of the pregnant uterus (Fig. 4).

**Figure 5:**
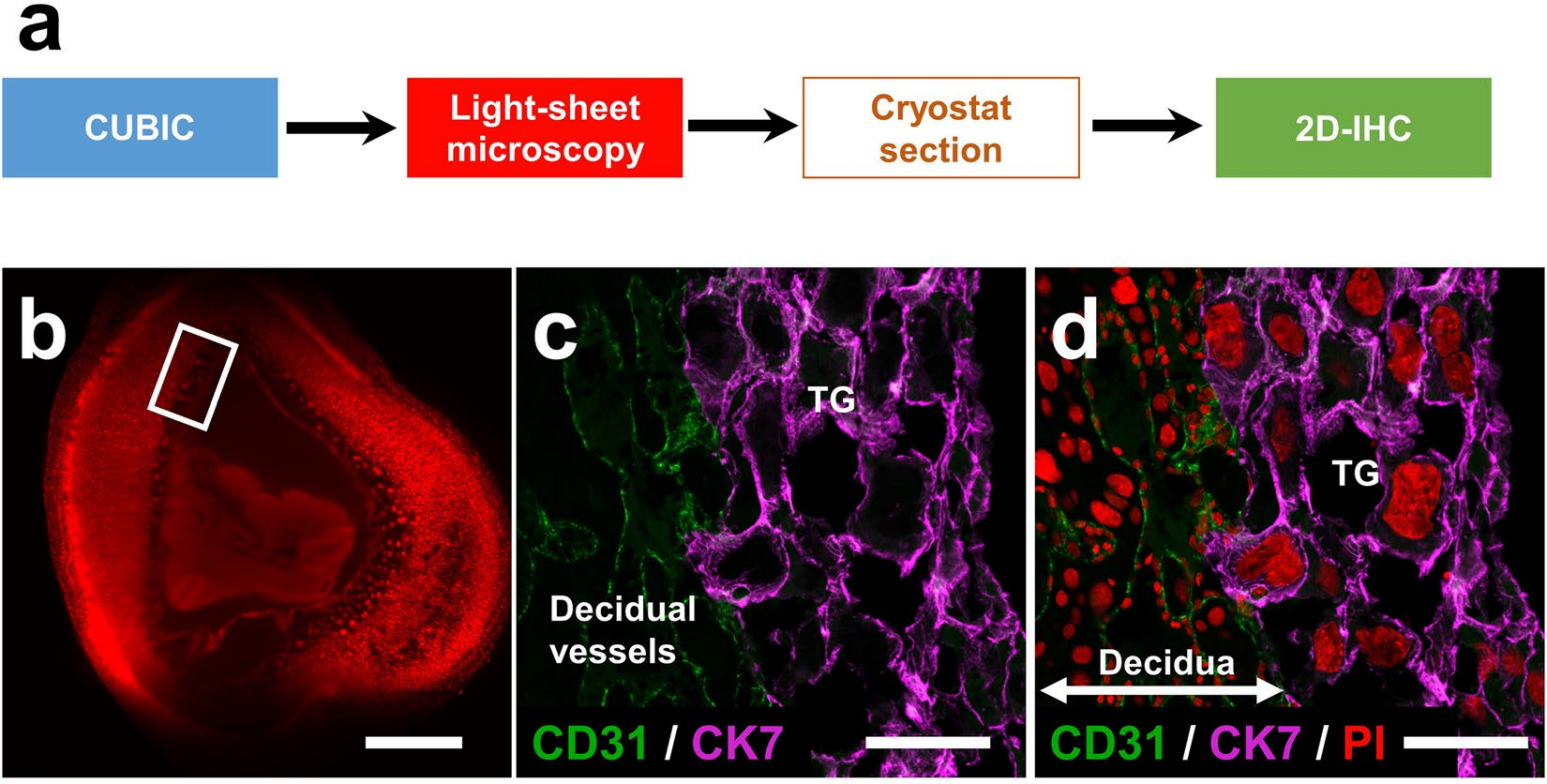
Immunohistochemical staining of tissue sections derived from the organ block of the transparent pregnant uterus. (**a**) The procedure of immunohistochemical staining of the tissue sections derived from the organ block of the transparent pregnant uterus. After the target sections, presented as a white square in the light-sheet image (**b**), were obtained with cryostat, immunohistochemical staining was performed with anti-CD31 (green) and anti-cytokeratin-7 (purple) antibodies (**c** and **d**). Note that trophoblast giant cells and decidual vessels are determinately visible at the feto-maternal interface. TG, trophoblast giant cells. Scale bars, 1 mm (**b**) and 100 µm (**c** and **d**).

In order to validate the histological characteristics of specific areas that were selected by the light-sheet microscope, further examination by immunohistochemical staining methods was performed after cutting new tissue sections from transparent organs (Fig. 5a). Using these tissues sections prepared from optimal sites, the expected fluorescence signals of a vascular endothelial marker, CD31, and trophoblast marker, cytokeratin-7, were successfully observed in the decidual region (Fig. 5c,d). These results also indicate that the antigenic reactivity of several molecules is preserved even after CUBIC procedures.

### 3D image of the EGFP-positive conceptus in the EGFP-negative pregnant uterus

**Figure 6:**
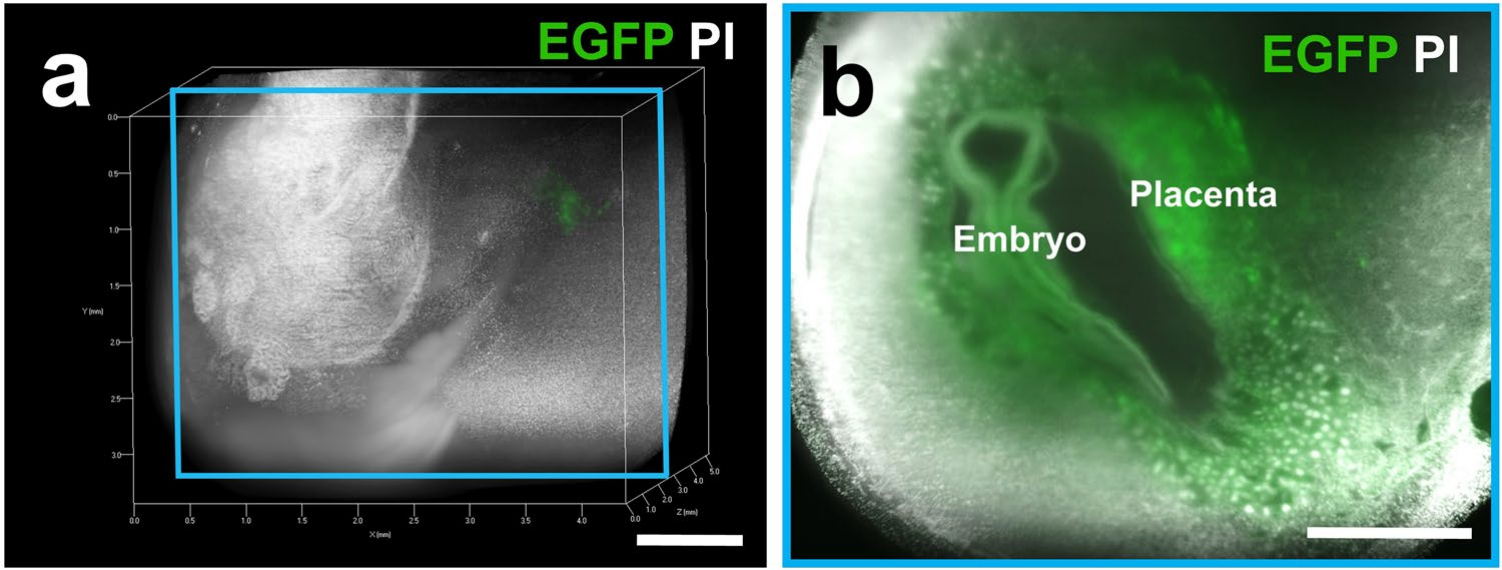
Three-dimensional image of the EGFP-positive conceptus in the uterus of wild-type mice. The EGFP-positive conceptus in the EGFP-negative uterus of wild-type mice was created by mating CAG-EGFP male mice with wild-type ICR female mice. Pregnant mice were transcardially perfused with 4% PFA and PI solution at E10.5. After the isolated reproductive organs were subjected to CUBIC, 3D and cross-sectional images of the EGFP-positive conceptus in the EGFP-negative uterus of wild-type mice were taken using a light-sheet microscope. A whole 3D image (**a**) and a sectional image within a blue square of the left panel (**b**) are shown. Note that fluorescence was clearly visible in the EGFP-positive conceptus, including the placenta and embryo. Scale bars, 1 mm.

**Figure 7:**
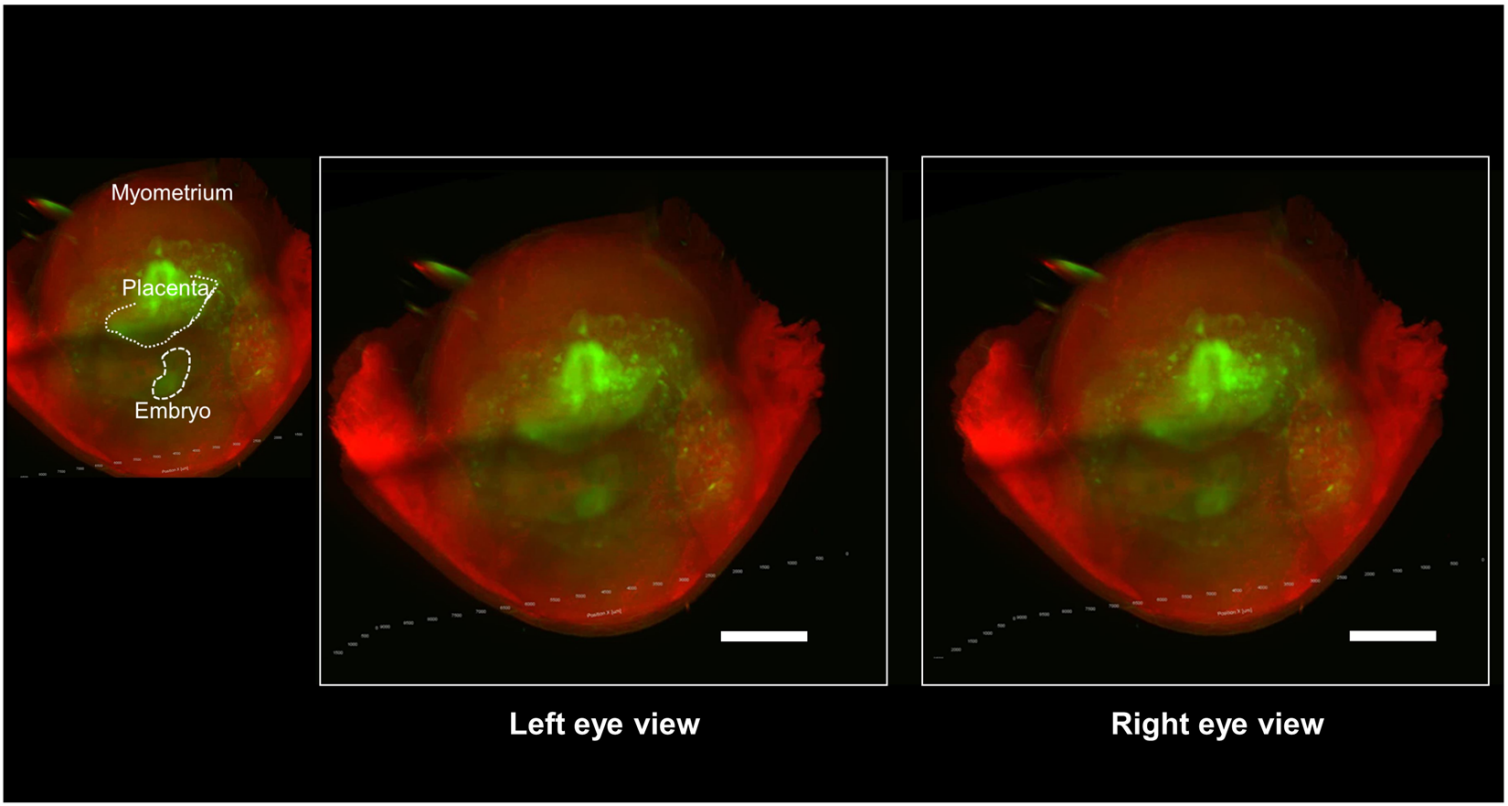
Stereoscopic image of the EGFP-positive conceptus in the EGFP-negative uterus of wild-type mice. The EGFP-positive conceptus in the EGFP-negative uterus of wild-type mice was created by mating CAG-EGFP male mice with wild-type ICR female mice. Pregnant mice were transcardially perfused with 4% PFA and PI solution at E10.5. After the isolated reproductive organs were subjected to CUBIC, 3D images were taken using a light-sheet microscope, and stereoscopic images were reconstructed electronically. Scale bars, 1 mm.

**Figure 8:**
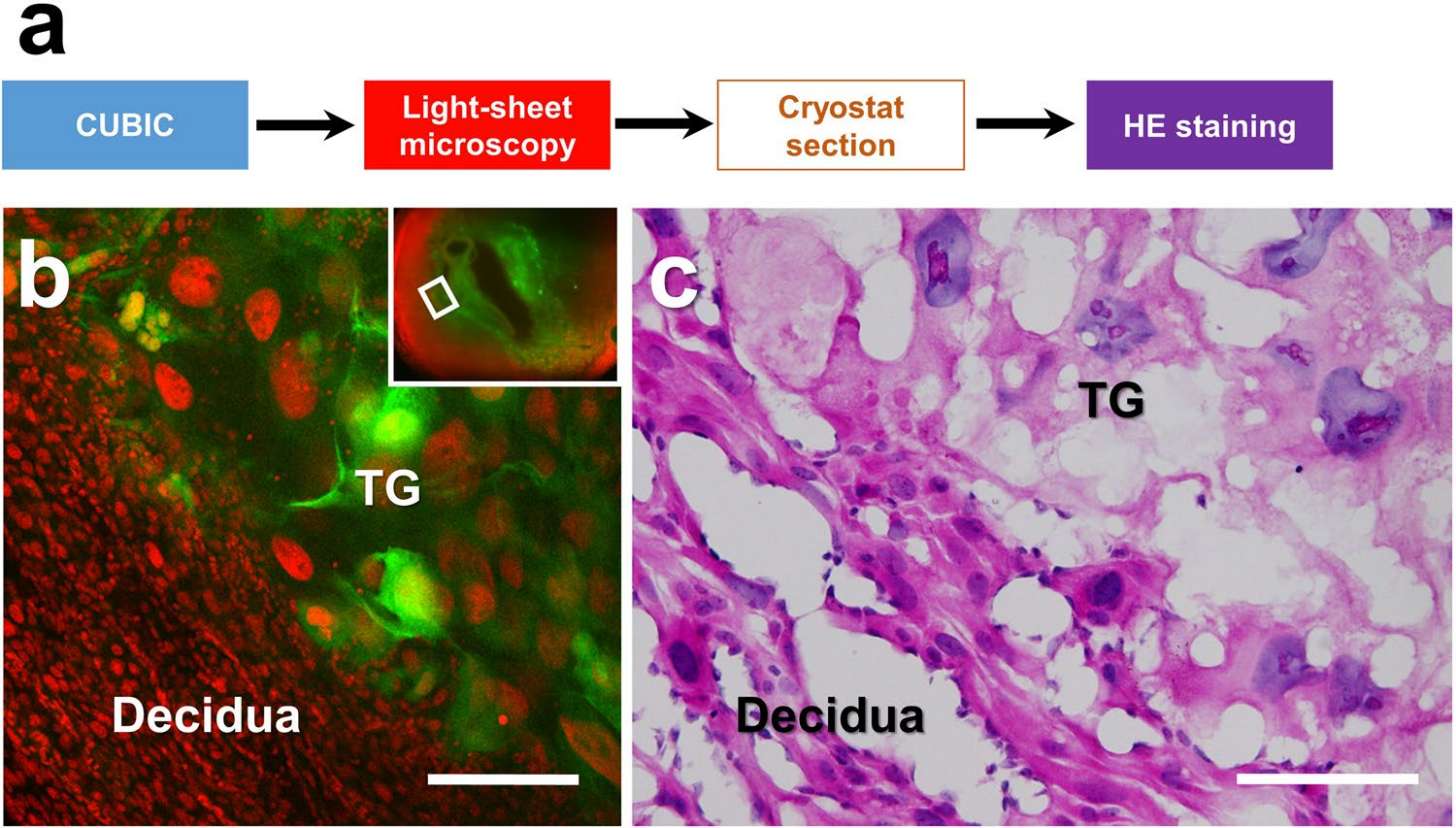
Histological analysis of transparent organ blocks by preparing the corresponding tissue slices. (**a**) The procedure of hematoxylin-eosin staining of the tissue sections derived from the organ block of the transparent EGFP-negative pregnant uterus containing the EGFP-positive conceptus. (**b**) Magnified images of the target area were obtained with a light-sheet microscope. After making tissue sections with a cryostat, hematoxylin-eosin staining was performed (**c**). Note that each cellular hematoxylin-eosin staining pattern was preserved even after CUBIC treatment. TG, trophoblast giant cells. Scale bars, 200 µm (**b**) and 100 µm (**c**).

Although nuclear labeling by PI clearly showed the cell distribution, this information alone was insufficient to recognize the cellular shape and/or histological structure of the organs. Therefore, we used transgenic mice expressing EGFP under the control of the CAG promoter, which contains the chicken β-actin promoter and cytomegalovirus enhancer. To further distinguish the invading trophoblasts and maternal tissues, we created an EGFP-positive conceptus in the EGFP-negative uterus by mating CAG-EGFP male mice with wild-type female mice in order to electively label the conceptus with EGFP. After transcardial perfusion with 4% PFA and PI solutions, the uterine tissues were isolated from pregnant mice at E10.5. The uterus was then subjected to CUBIC, and 3D images were taken using a light-sheet microscope. The EGFP-positive embryo and placenta were clearly observed within the EGFP-negative uterus in 2D images of X-Y cross-sections (Fig. 6b). Their sequential 2D images are demonstrated as supplemental video data (Supplementary Movie S1). Moreover, using a stereoscopic image, the 3D location of the EGFP-positive trophoblast giant cells was clearly detected throughout the EGFP-negative uterine wall, which are distributed in the peripheral region to chorioamniotic membrane and in close contact with the decidua (Fig. 7 and Supplementary Movie S2). These cells can be further analyzed by hematoxylin-eosin staining using the corresponding tissue sections that were newly produced from the transparent tissue blocks (Fig. 8a). By this procedure, trophoblast giant cells with low-level eosinophilic cytoplasm were clearly observed and the precise spatial relationship between embryo-derived trophoblast giant cells and maternal decidual cells with eosinophilic cytoplasm could be pathologically evaluated using a standard staining method together with the whole structures of the surrounding tissues (Fig. 8c). This may offer a marked advantage on adopting a clinico-pathological approach.

## Discussion

Here, we show that a whole 3D image of the murine pregnant uterus and conceptus can be successfully obtained by combining a tissue clearing method and light-sheet microscopy. To our knowledge, this is the first report to demonstrate stereoscopic transparent images of reproductive organs including late stage of pregnant uterus.

Previous pioneering studies demonstrated two key factors of tissue clearing: removal of lipids and homogenization of refractive indices^18^. Most tissue clearing methods have been successfully applied to the brain, which is a lipid-rich organ^11–15^. However, these methods are not always effective for other organs containing large amounts of blood or muscles because light absorbance by endogenous chromophores, mainly hemoglobin and myoglobin, interferes with the transparency of the organs^15^. Although the uterus does not contain high level of lipids, it has a large amount of muscle. In addition, the placenta is a blood-rich organ. Consequently, we chose the CUBIC method that efficiently removes heme without disrupting fluorescence proteins. By applying long-term incubation with the CUBIC-1 reagent, we successfully made the pregnant uterus including the conceptus transparent (Fig. 1). Previous reports using other organs pointed out that tissue clearing often causes marked changes in the volume of samples. Fortunately, the volume and fine architecture of the pregnant uterus were preserved even after applying CUBIC. From these findings, we conclude that a CUBIC-based protocol is one of the effective tissue clearing methods for the pregnant uterus to enable transparent 3D images of intrauterine structures to be obtained.

To label the nuclei of the transparent tissues, we used PI staining. As shown in a previous report, we confirmed that fluorescent signals of PI were not eliminated by CUBIC treatment. Since we could not obtain clear PI staining of the nuclei in the deep sites of the uterus by immersion in PI solutions alone, we added PI to 4%-PFA fixative solution. By this modification, we obtained clear PI signals in the deep sites of the uterus, indicating that transcardial perfusion of PI solutions is useful for labeling the nuclei in the deep tissues (Fig. 3). However, PI signals of the embryos in the pregnant uteri were sometimes faint even when their signals were analyzed by a highly sensitive light-sheet microscope. Additional improvement of the methodology is necessary in the future.

Based on PI signals, we successfully constructed 3D images using a light-sheet microscope and obtained 3D images from all directions (Fig. 3). From these pictures, stereoscopic images from any angle can be reconstructed electronically, which also enables us to analyze the 3D-positional relationships among the uterus, placenta, and embryo (Fig. 4). 3D images of intrauterine structures also provide more precise data on the volumes, numbers, and locations of various cells. More importantly, from these data, we can analyze free-angle images of cross-sections with single-cell resolution using a computer system (Fig. 3d and h). The conventional histological techniques require large numbers of thin tissue sections to reconstruct 3D images of whole organs. It is laborious and difficult work to prepare sequential thin tissue sections. In contrast, as shown in this study, the combination of tissue clearing techniques and light-sheet microscopy is a useful and relatively straightforward method. In addition, after determining an optimal cross-section angle in accordance with the purpose of each study, we can cut and prepare the corresponding tissue slices that are adequate for further examination such as immunohistochemistry (Fig. 5) and hematoxylin-eosin staining (Fig. 8). The above subsequent optional experiments using a transparent tissue block (Fig. 1) is one of the important advantages of this method.

To further obtain distinct images of the embryo and placenta, we used EGFP- transgenic mice. Based on positive green signals caused by EGFP, we identified the precise 3D-location of the trophoblast giant cells and the invading trophoblasts in the feto-maternal interface in the uterus (Fig. 6). The detection of EGFP in the embryo also helps us to distinguish the developing organs. These advantages made it possible to histologically analyze the special zone further by preparing the corresponding tissue slices (Fig. 8).

Although we did not perform further analysis in this study, we successfully obtained a transparent placental tissue block in the later phase of gestation (E14.5, Fig. 1e). This suggests that analysis based on 3D transparent visualization is also possible in the placental tissues in the later stage of placental development. In addition, a recent report showed 3D images of mouse uterine glands around implantation sites at E4.5 using the BABB tissue clearing method and confocal microscopy^19^. These findings suggest that tissue clearing methods are extremely useful as a way to investigate reproductive organs.

In conclusion, we successfully obtained 3D whole images of the murine uterus and intrauterine conceptus using a modified tissue clearing CUBIC method. By this procedure, we can stereoscopically recognize the 3D positional relationship of various tissues and analyze free-angle images of cross-sections with single-cell resolution. After selecting an optimal cross-section angle, the corresponding tissue slices can be applied to the subsequent immunohistochemical experiments. Furthermore, using transgenic mice, we can analyze the precise location of invading trophoblasts in the feto-maternal interface. From these findings, we conclude that this procedure is a useful tool to analyze placental and embryonic development from a new aspect, and propose that its application to other reproductive organs will contribute to clarifying pathophysiological mechanisms in reproductive functions.

## Methods

### Preparation of reagents

CUBIC reagents were prepared as previously described^15^. CUBIC-1 reagent was prepared as a mixture of 25% urea (Nacalai Tesque, 35904-45, Japan), 25% N,N,N′,N′-tetrakis (2-hydroxypropyl) ethylenediamine (Tokyo Chemical Industry, T0781, Japan), and 15% polyethylene glycol mono-pisooctylphenyl ether (Triton X-100) (Nacalai Tesque, 25987-85, Japan). CUBIC-2 reagent was prepared as a mixture of 50% sucrose (Nacalai Tesque, 30403-55, Japan), 25% urea, 10% 2,20,20′-nitrilotriethanol (Wako, 145-05605, Japan), and 0.1% Triton X-100. Both reagents were prepared just prior to use. Before adding Triton X-100, all other chemicals were dissolved with a hot stirrer. Because water evaporation will make it difficult for highly concentrated chemicals to be dissolved, the weight of the solution was monitored frequently, and any water that had evaporated was replenished during a mixing step. After all chemicals except Triton X-100 had been dissolved, the solution was cooled to room temperature, and finally Triton X-100 was added.

### Animals

We used transgenic mice expressing EGFP under the control of the CAG promoter (C57BL/6-Tg)^20^ and wild-type female ICR mice. ICR mice were purchased from SLC (Hamamatsu, Japan), and all mice were reared on a normal 12-hour light/dark schedule. The day of conception was counted as embryonic day 0 (E0). The EGFP-positive conceptus in the uterus of wild-type mice was created by mating CAG-EGFP male mice with wild-type ICR female mice. All experimental procedures and housing conditions were approved by the Kanazawa University Animal Care Committee, and all of the animals were treated in accordance with the Institutional Guidelines for Experiments Using Animals.

### The CUBIC protocol for pregnant uterus

CUBIC was performed as described previously with modifications (Fig. 1a)^15^. After deep anesthesia with pentobarbital, pregnant mice were fixed by transcardial perfusion using 4% paraformaldehyde (PFA)/PBS and propidium iodide (PI, Life Technologies, 10 mg/mL) solution, and then the uteri were isolated. Isolated uteri were further immersed in 4% PFA at 4 °C overnight. Then, the fixed organs were immersed in CUBIC-1 reagent at 37 °C for 3 days with gentle shaking. After the CUBIC-1 reagent was changed, the organ was immersed for 2 additional days. The organ was washed with PBS 3 times at room temperature with gentle shaking, immersed in 20% sucrose in PBS for one day, and immersed in CUBIC-2 regent for 2 days. For immersion staining with PI, 10 mg/ml PI was added to the CUBIC-1 reagent.

### Microscopy and image analysis

Bright field images of the pregnant uterus and detached placenta were taken using a stereomicroscope (MZ16F, Leica). Immunohistochemically stained tissue sections were observed with an epifluorescence confocal microscope (LSM510, Carl Zeiss). Three-dimensional images of transparent organs were acquired using a light-sheet microscope (Lightsheet Z.1, Carl Zeiss). Images of whole reproductive organs were obtained using a 5x/0.16 NA objective lens, and detailed single-cell resolution images were acquired using a 20x/1.0 NA objective lens for the clearing method. Three-dimensional images were analyzed using ZEN software (Carl Zeiss). An excitation wavelength of 638 nm was used for visualizing autofluorescence signals.

### Immunohistochemical staining of tissue sections derived from the organ block of the transparent pregnant uterus

Immunostaining was performed as described previously with slight modifications^21, 22^. Pregnant uteri were subjected to CUBIC and analyzed with a light-sheet microscope. To prepare tissue sections, the transparent pregnant uteri were washed with PBS 3 times at room temperature with gentle shaking, cryoprotected by overnight immersion in 20% sucrose in PBS, and embedded in OCT compound. Tissue sections of a 14-μm thickness were made using a cryostat, permeabilized with 0.5% Triton X-100 in PBS, and incubated at 4 °C overnight with rat anti-CD31 antibody (1:500, BD Pharmingen) and rabbit anti-cytokeratin-7 antibody (1:1000, Abcam). After being incubated with Alexa488- or Alexa647-conjugated secondary antibody at 37 °C for 2 hours, the sections were washed and mounted.

## Electronic supplementary material

Supplementary information

Supplementary Movie S1

Supplementary Movie S2
