# Supplementary material for: Three-dimensional visualization of intrauterine conceptus through the uterine wall by tissue clearing method
Source: Sci Rep. 2017 Jul 20;7:5964. doi: 10.1038/s41598-017-06549-6 (PMC5519638; doi:10.1038/s41598-017-06549-6)
Supplement: Supplementary file 1 — Supplementary information [file 41598_2017_6549_MOESM1_ESM.pdf]

Three-dimensional visualization of intrauterine conceptus through the uterine wall by tissue clearing method

Kyosuke Kagami[1,2], Yohei Shinmyo[2], Masanori Ono[1], Hiroshi Kawasaki[2,3], and Hiroshi Fujiwara[1]

[1] Department of Obstetrics and Gynecology, Graduate School of Medical Sciences, Kanazawa University, Ishikawa 920-8640, Japan
[2] Department of Medical Neuroscience, Graduate School of Medical Sciences, Kanazawa University, Ishikawa 920-8640, Japan
[3] Brain/Liver Interface Medicine Research Center, Kanazawa University, Ishikawa 920-8640, Japan

Address correspondence to
Hiroshi Fujiwara, MD, PhD
Department of Obstetrics and Gynecology, Graduate School of Medical Sciences, Kanazawa University, Takara-machi 13-1, Kanazawa, Ishikawa 920-8640, Japan
Tel.: +81-76-265-2425, Fax: +81-76-234-4266
E-mail: fuji@kuhp.kyoto-u.ac.jp : fuji@med.kanazawa-u.ac.jp

Hiroshi Kawasaki, MD, PhD
Department of Medical Neuroscience, Graduate School of Medical Sciences, Kanazawa University, Takara-machi 13-1, Kanazawa, Ishikawa 920-8640, Japan
Tel.: +81-76-265-2363, Fax: +81-76-234-4728, E-mail: hiroshi-kawasaki@umin.ac.jp

Key words: CUBIC, 3D visualization, placenta, uterus, tissue clearing

Supplementary Movie S1. Sequential 2D images of X-Y cross-sections of the EGFP-positive conceptus in the EGFP-negative uterus of wild-type mice.

The EGFP-positive conceptus in the EGFP-negative uterus was created by mating CAG-EGFP male mice with wild-type ICR female mice. Pregnant mice were transcardially perfused with 4% PFA and PI solution at E10.5. After the isolated pregnant uterus was subjected to CUBIC, sequential 2D images of X-Y cross-sections were taken using a light-sheet microscope.

Supplementary Movie S2. A 3D image of the EGFP-positive conceptus in the EGFP-negative uterus of wild-type mice.

The EGFP-positive conceptus in the EGFP-negative uterus was created by mating CAG-EGFP male mice with wild-type ICR female mice. Pregnant mice were transcardially perfused with 4% PFA and PI solution at E10.5. After the isolated pregnant uterus was subjected to CUBIC, 3D images were taken using a light-sheet microscope.
